# Comparative genomic analysis of ten clinical *Streptococcus pneumoniae* collected from a Malaysian hospital reveal 31 new unique drug-resistant SNPs using whole genome sequencing

**DOI:** 10.1186/s12929-018-0414-8

**Published:** 2018-02-15

**Authors:** Hassan Mahmood Jindal, Babu Ramanathan, Cheng Foh Le, Ranganath Gudimella, Rozaimi Razali, Rishya Manikam, Shamala Devi Sekaran

**Affiliations:** 1 Department of Medical Microbiology, University of Malaya, 50603 Kuala Lumpur, Malaysia; 2 Department of Biological Sciences, School of Science and Technology, Sunway University, 47500 Kuala Lumpur, Malaysia; 3 School of Pharmacy, University of Nottingham Malaysia Campus, 43500 Semenyih, Selangor Malaysia; 4 Sengenics HIR, University Malaya, 50603 Kuala Lumpur, Malaysia; 5 Department of Trauma and Emergency, University Malaya Medical Centre, 50603 Kuala Lumpur, Malaysia; 6 Department of Microbiology, Faculty of Medicine, MAHSA University, 42610 Jenjarom, Malaysia

**Keywords:** *Streptococcus Pneumoniae*, Antibiotic-resistance, Whole genome sequencing, Single nucleotide polymorphism, Penicillin-binding proteins

## Abstract

**Background:**

*Streptococcus pneumoniae* or pneumococcus is a leading cause of morbidity and mortality worldwide, specifically in relation to community-acquired pneumonia. Due to the overuse of antibiotics, *S. pneumoniae* has developed a high degree of resistance to a wide range of antibacterial drugs.

**Methods:**

In this study, whole genome sequencing (WGS) was performed for 10 clinical strains of *S. pneumoniae* with different levels of sensitivity to standard antibiotics. The main objective was to investigate genetic changes associated with antibiotic resistance in *S. pneumoniae*.

**Results:**

Our results showed that resistant isolates contain a higher number of non-synonymous single nucleotide polymorphisms (SNPs) as compared to susceptible isolates. We were able to identify SNPs that alter a single amino acid in many genes involved in virulence and capsular polysaccharide synthesis. In addition, 90 SNPs were only presented in the resistant isolates, and 31 SNPs were unique and had not been previously reported, suggesting that these unique SNPs could play a key role in altering the level of resistance to different antibiotics.

**Conclusion:**

Whole genome sequencing is a powerful tool for comparing the full genome of multiple isolates, especially those closely related, and for analysing the variations found within antibiotic resistance genes that lead to differences in antibiotic sensitivity. We were able to identify specific mutations within virulence genes related to resistant isolates. These findings could provide insights into understanding the role of single nucleotide mutants in conferring drug resistance.

**Electronic supplementary material:**

The online version of this article (10.1186/s12929-018-0414-8) contains supplementary material, which is available to authorized users.

## Background

*Streptococcus pneumoniae* is a Gram–positive human pathogen naturally inhabiting the human nasopharynx (which considered to be the reservoir as this pathogen has no animal or insect vectors) and is responsible for invasive and noninvasive diseases including meningitis, pneumonia, bacteremia, otitis media, and sinusitis [1–3]. According to the World Health Organization (WHO), this bacterium is responsible for 1.6 million deaths annually, including 0.7–1 million in children less than 5 years old and mostly in developing countries [4, 5]. In the United States, the annual number of deaths caused by pneumococcal pneumonia or meningitis is 40,000 [6, 7]. In Asia, *S. pneumoniae* is the major cause of acute respiratory infections (ARIs) in children under 5 years old [8]. Five of the 10 countries with the largest number of deaths caused by pneumococcal infections in children below 5 years old are located in Asia, including India, China, Bangladesh, Pakistan, and Afghanistan [9]. Currently, more than 93 different *S. pneumoniae* serotypes have been identified based on the immunochemical differences in the capsular polysaccharides [9–11].

The mechanism used by *S. pneumoniae* to become pathogenic is still poorly understood, and most likely it depends on the interaction between pneumococcal virulence factors and the host’s immunological response [12, 13]. For decades, penicillin has been the principle option for the treatment of infections associated with *S. pneumoniae* [14]*.* The main targets for penicillin and other β-lactams antibiotics are the penicillin-binding proteins (PBPs). These enzymes are essential for the synthesis of the bacterial cell wall. β-lactams act by binding to these enzymes and reducing peptidoglycan synthesis and remodeling. Subsequently, this leads to disruption of cell wall integrity and cell lysis [15, 16].

Like other Gram-positive bacteria, *S. pneumoniae* has developed significant resistance over the last few decades against a wide range of antibiotics due to extensive over-use. Mutating the target proteins such as PBPs to reduce their affinity to β-lactam antibiotics is the main resistance mechanism developed by pneumococci to resist β-lactams [17]. Moreover, *S. pneumoniae* has developed powerful resistance tools against erythromycin and other macrolides by modifying the target site (23S ribosomal RNA) using the *erm* gene or by efflux of the antibiotic from the bacterial cell through acquisition of the *mef* gene [18]. Tetracyclines are bacteriostatic agents that stop bacteria from reproducing by binding to the 30S subunit of the bacterial ribosome. Pneumococcal resistance to tetracycline occurs through ribosomal protection mediated by *tet*(O) and *tet*(M) genes [19]. There has been a huge increase in the number of penicillin-resistant pneumococcal isolates over the past three decades, and many strains are now resistant against common antibacterial drugs such as β-lactams, macrolides, and fluoroquinolones [20, 21]. A study conducted by Hackel and his co-workers on 2173 worldwide pneumococcal isolates, showed that 33.3% of the isolates were resistant to penicillin, 22.9% to erythromycin, and 16.2% to both erythromycin and penicillin [22].

Whole genome sequencing (WGS) has become a powerful tool for drug development by allowing researchers to investigate the mode of action of antibiotics and the mechanisms involved in bacterial resistance [23, 24]. Additionally, WGS can be utilised to investigate the evolution of resistance in real-time under a range of conditions [25]. In this study, we report the whole genome sequencing for 10 pneumococcal isolates with a range of susceptibility and resistance to different antimicrobial drugs to elucidate the association between antibiotic resistance and the underlying genetic changes.

## Methods

### Bacteria and MIC determinations

Ten pneumococcal clinical strains were collected from the microbiological specimens of patients cared for at University of Malaya Medical Centre (UMMC) over a three-year period from September 2010 to May 2012 (Table 1). All the isolated strains were stored in brain heart infusion (BHI) broth at − 80 °C. Pneumococcal isolates were grown in blood agar containing 5% defibrinated sheep blood as previously described [26]. Cultures were incubated for 16–24 h at 37 °C under 5% CO_2_. Multiplex PCR was performed to identify each strain serotype as previously described [27]. Minimal inhibitory concentration (MIC) was determined following the broth microdilution assay as described by the Clinical and Laboratory Standards Institute (CLSI) guidelines. Cation-adjusted Mueller–Hinton broth with lysed horse blood was inoculated with a 5 × 10^5^ cfu/mL bacterial suspension. The MIC was recorded as the lowest dilution showing no visible growth. All of the results were obtained from three independent trials.

**Table 1:** Bacterial strains and sources used for the genomic comparison of *S. pneumoniae* strains

Isolate	Isolation date	Sex	Source	Serotype^a^
SPS1	15/9/2010	NA	Nasopharyngeal swab	NT
SPS2	21/5/2011	Female	Nasopharyngeal swab	1
SPS3	21/5/2011	Male	Nasopharyngeal swab	19F
SPS4	20/2/2012	Female	Nasopharyngeal swab	14
SPS5	16/3/2012	Female	Swab from eye	23F
SPS6	18/5/2012	Male	Nasopharyngeal swab	15B/C
SPS7	9/5/2011	Male	Blood	1
SPS8	8/3/2011	Female	Nasopharyngeal swab	14
SPS9	26/4/2011	Male	Blood	18
SPS10	10/5/2011	Male	Blood	8

^a^all serotypes were identified using multiplex PCR as described before

(Pai et al., [27])

*Abbreviations*: *NA* not available, *NT* non-typeable

### Library preparation and whole genome sequencing

A DNeasy Blood & Tissue Kit (Qiagen) was used to extract genomic DNA from pneumococcal cells cultured overnight following the manufacturer’s guidelines. Whole genome sequencing was performed using the Illumina HiSeq 2000 platform consisting of 1 lane 100 bp paired-end reads. Briefly, Covaris S2 was used to fragment all genomic DNA at the temperature of 5.5 to 6 °C for 40 s. The fragmented DNAs were ends repaired, added with dA base and ligated with Illumina indexed adapters. Invitrogen 2% agarose E-gel was used for size selections of the samples. The selected DNA fragments with adapter molecules on both ends underwent ten cycles of PCR for amplification of prepared material. The samples were then diluted to 10 nM and pooled together. The libraries were loaded onto one lane of Illumina HiSeq 2000 flow cell v3 for sequencing. Illumina adapter sequences were trimmed on both ends of the reads which resulted in low quality bases on the 5′ end of the reads. Low quality bases were removed with a quality score filter of ≥ 30 using PRINSEQ version 0.20.3 [28].

### Assembly

Assembly was performed utilising SPAdes assembler version 3.8.1 [29] by using metaSPAdes option specific for metagenome assemblies. Assembler was run using iterative kmer lengths ranging from 27 to 77. The 10 assembled genomes were compared to the reference genome *S. pneumoniae* TIGR4 (NC_003028.3) using MetaQuast [30].

### Gene prediction and clustering

Genes prediction on the draft assemblies was performed by using the Prokka (Prokaryotic annotation) tool. Functionally, Prokka predicts genes based on available annotation information such as CDS and proteins. It builds HMM databases which are searched by using HMMER3. Prokka was run using customised parameters that were set to annotate against reference genome *S. pneumoniae* TIGR4 with an evalue of 1e-10. To create gene clusters among 10 isolates and the reference genome all the amino acid sequences in fasta format were retrieved. All proteins were subjected for BLASTp (e.value <1e-5) against the same set of sequences in order to perform all versus all blast. A connection (edge) between two genes was assigned if more than one third of the region aligned to both genes. An h-score (0 to 100) was used to weight the similarity (edge). For two genes G1 and G2, the Hscore was defined as score (G1G2) / max (score (G1G1), score (G2G2); the score used here was the BLAST raw score. Gene families were identified by using clustering by Hcluster_sg [31]. We used the average distance for the hierarchical clustering algorithm, with the parameters of minimum edge weight set to 5 and the minimum edge density (total number of edges / theoretical number of edges) set to 0.35.

### Variant calling and phylogeny

Single nucleotide polymorphisms (SNPs) were identified using kSNP3 program (version 3.021) which identifies pan-genome SNPs in a set of genome sequences and builds a phylogenetic tree based on the SNPs [32]. kSNP3 was run using the standard mode of SNP detection and annotation using *S. pneumoniae* TIGR4 as reference with Kmer size of 11. Kmer was calculated by Kchooser program which accurately defines a kmer size based on the draft genome assemblies. Phylogeny trees are parsimony trees based on consensus trees from different samples. Although parsimony trees do not define evolution lineage, they do help to define the nearer samples based on changes per number of SNPs. A complex heat map package from Bioconductor was used to generate heat maps in R. Clusters are predicted using Euclidean distance method.

### Statistical analysis

Statistical analysis testing the difference in SNP number between the antibiotic resistant and susceptible isolates was performed using two-sample Student’s t-test with a significant level at *p* < 0.05.

## Results

### Selection and whole-genome sequencing of *S. pneumoniae*

Ten isolates were selected from a larger collection of pneumococcal isolates according to their susceptibility to four different antibiotics: penicillin, cefotaxime, erythromycin, and tetracycline. Table 2 summarises the MICs for all 10 isolates. Isolates SPS1, SPS2, and SPS3 were non-susceptible to all antibiotics; isolate SPS4 was susceptible to penicillin, cefotaxime, and erythromycin; SPS5 exhibited susceptibility to cefotaxime and erythromycin, but it showed resistance to penicillin and tetracycline. Isolate SPS6 was susceptible to all four antibiotics; conversely, isolates SPS7 and SPS10 were resistant to all four antibiotics. Isolates SPS8 and SPS9 were resistant to all antibiotics but they showed susceptibility to penicillin.

**Table 2:** Antibiotic susceptibility profiles of *S. pneumoniae* isolates

Isolate ^a^	MIC (μg/ml)^b^			
	PEN ^c^	CTX ^c^	ERY ^c^	TET ^c^
SPS1	2	1	2	16
SPS2	2	1	> 2	> 16
SPS3	4	1	> 2	> 16
SPS4	0.06	≤0.063	≤0.016	> 16
SPS5	1	0.125	0.031	4
SPS6	0.06	≤0.063	≤0.016	≤0.125
SPS7	2	2	> 2	> 16
SPS8	0.5	> 8	2	> 16
SPS9	0.25	8	2	16
SPS10	2	2	> 2	16

^a^Isolates SPS1, SPS2, and SPS3 are non-susceptible to all antibiotics. Isolate SPS4 is susceptible to penicillin, cefotaxime, and erythromycin, but resistant to tetracycline. SPS5 is susceptible to cefotaxime and erythromycin, but resistant to penicillin and tetracycline. SPS6 is susceptible to all four antibiotics. SPS7 and SPS10 are resistant to all four antibiotics, SPS8 and SPS9 were resistant to all antibiotics except penicillin

^b^*MIC* Minimum inhibitory concentration

^c^*PEN* Penicillin, *CTX* Cefotaxime, *ERY* Erythromycin, *TET* Tetracycline

**Fig. 1:**
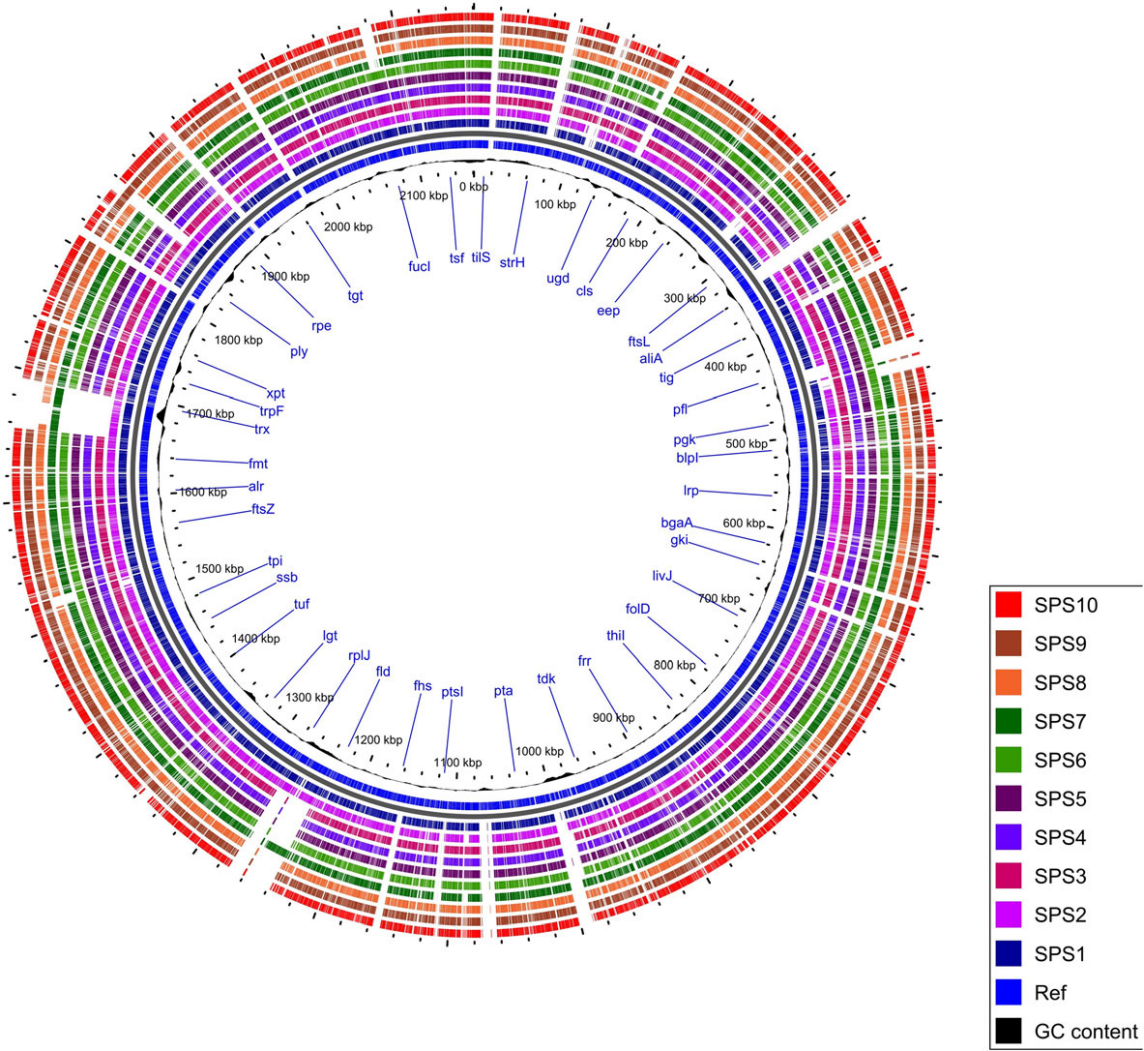
Circular genome map of 10 *S. pneumoniae* isolates compared to reference genome TIGR4. Rings from the outside inward: SP10, SP09, SP08, SP07, SP06, SP05, SP04, SP03, SP02, SP01, and reference genome *S. pneumoniae* TIGR4 (NC_003028.3). The blank spaces in the rings represent matches with less than 70% or no BLAST matches to the reference genome. The image was prepared using Blast Ring Image Generator [51]

The WGSs of all 10 isolates were conducted to investigate the association between antibiotic-resistance and the underlying genomics variations. The genomic DNA of all the isolates was sequenced using the Illumina HiSeq 2000 platform. The draft genome assemblies for the 10 isolates have been submitted to the NCBI BioProject under the project accession number PRJNA317517 (http://www.ncbi.nlm.nih.gov/bioproject/317517). The sequencing consisted of one lane 100 bp paired-end reads, yielding approximately 0.6Gbp to 3.6Gbp for *S. pneumonia*e. More than 80% of the reads were above a Phred quality score of 30 indicating the high-quality of the sequencing data. The overall GC% content for all 10 isolates ranged from 39.12 to 39.72% and was similar to that of the TIGR4 reference genome (39.7%) [33]. The number of genes was 2352 and 2159 for SPS1 and SPS2, respectively. SPS3, SPS4, SPS5, and SPS6 had gene contents of 1983, 1983, 2020, and 1984, respectively. SPS7, SPS8, SPS9, and SPS10 showed gene contents of 2064, 1980, 2035, and 1924, respectively (Additional file 1). The number of tRNAs was also similar for all the 10 isolates in the range of 41-46 (Additional file 1). Figure 1 represents a circular map of the ten pneumococcal clinical isolates compared to the reference genome of isolate TIGR4. All 10 isolates showed > 90% identity with the reference genome. Different colours represent the BLASTn matches between 70% to 100% nucleotide identities. The full assembly and gene content for each pneumococcal isolate can be found in Additional file 1.

### Core genome polymorphism

**Fig. 2:**
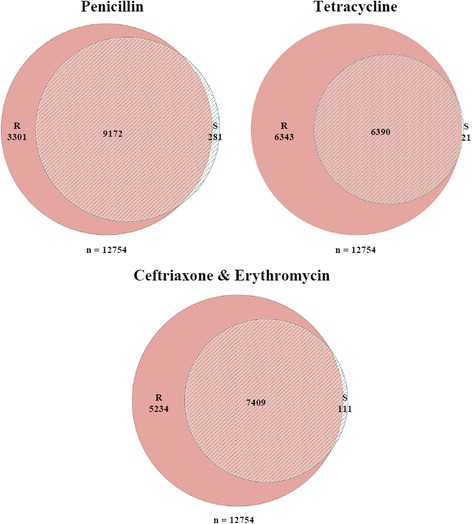
The Venn diagram summarizes the number of SNPs among the resistant and susceptible isolates for each antibiotic. PEN (Penicillin), CTX (Cefotaxime), ERY (Erythromycin), and TET (Tetracycline)

To identify sequence variations, WGS reads from each strain were mapped to the TIGR4 reference genome of *S. pneumoniae* using the Bowtie2 software [34]. The genome sequences of all the clinical isolates revealed a high level of similarity and the virulence genes that are known to be involved in drug-resistance are well conserved among all the 10 isolates (Table 3). In order to identify differences that alter the level of resistance of these clinical isolates we focused on identifying SNPs in genes engaged with antibiotics pathways. Table 4 represents the total number of SNPs identified for each isolate, which ranged from 3600 to 6548 SNPs. SNPs that cause a change in amino acids, start codons, and stop codons were classified as “non-synonymous SNPs”. Figure 2 illustrates the distribution of all SNPs in both antibiotic resistant and susceptible isolates. The majority of these non-synonymous SNPs associated with pneumococcal essential genes were present in antibiotic resistant strains (*p* = 0.016). Penicillin-resistant isolates showed 3301 SNPs, while susceptible isolates had only 281 SNPs. 6343 SNPs were associated with tetracycline-resistant isolates compared to only 21 SNPs associated with isolate SPS6 (Fig. 2). Similarly, ceftriaxone and erythromycin resistant isolates showed greater number of SNPs (5234) compared to 111 SNPs in susceptible isolates (Fig. 2). The complete list of SNPs in the 10 isolates sequenced and the TIGR4 reference genome can be found in Additional file 2. To explore the potential link between sequence variants with virulence characteristics, non-synonymous polymorphisms were extracted from genes annotated as virulence factors or involved in bacterial resistance [35, 36] (Table 4). The conserved non-synonymous polymorphisms in all resistant pneumococcal isolates were identified. These SNPs could possibly play an important biological role as they result in stop codons or frame shifts in protein sequence (Table 5). A total of 16 genes with 90 non-synonymous SNPs found only in the resistant isolates were identified. The presence of some of these SNPs in more than one resistant isolate suggests that these SNPs or a subset of them might have potential roles in antibiotic resistance. For example, the same mutation (G597E) associated with penicillin binding protein PBP2b was found in four different resistant isolates SPS7, SPS8, SPS9, and SPS10. Similarly, mutation (T23A) associated with virulent gene pneumococcal surface protein A (PspA) was identified in four resistant isolates SPS1, SPS7, SPS8, and SPS9 (Additional file 3). By blasting our sequences, we were able to identify 31 unique non-synonymous SNPs associated with penicillin binding proteins (PBPs) and other virulent genes that were not previously published (Table 6).

**Table 3:** Presence and absence of genes involved in virulence and antibiotic-resistance in each of the 10 clinical isolates

Locus name^a^	Gene name and/or description	Presence of virulent genes in Pneumococcal isolates									
		SPS01	SPS02	SPS03	SPS04	SPS05	SPS06	SPS07	SPS08	SPS09	SPS10
SP_0531	blpI; bacteriocin	1	1	0	0	0	0	0	0	1	0
SP_0041	blpU; bacteriocin	1	1	1	1	1	1	1	1	0	1
SP_0109	bacteriocin	1	1	0	0	0	0	1	1	1	1
SP_0544	immunity protein BlpX	1	1	0	0	0	0	1	1	0	1
SP_1315	V-type ATP synthase subunit D	1	1	0	0	0	0	1	0	0	0
SP_1318	V-type ATP synthase subunit F	1	1	0	0	0	0	1	0	0	0
SP_1319	V-type ATP synthase subunit C	1	1	0	0	0	0	1	0	0	0
SP_1320	V-type ATP synthase subunit E	1	1	0	0	0	0	1	0	0	0
SP_1321	V-type ATP synthase subunit K	1	1	0	0	0	0	1	0	0	0
SP_1322	V-type ATP synthase subunit I	1	1	0	0	0	0	1	0	0	0
SP_0346	cpsA; capsular polysaccharide biosynthesis protein	1	1	1	1	1	1	1	1	1	1
SP_0347	cpsB; capsular polysaccharide biosynthesis protei	1	1	1	1	1	1	1	1	1	1
SP_0348	cpsC; capsular polysaccharide biosynthesis protein	1	1	1	1	1	1	1	1	1	1
SP_0349	cpsD; capsular polysaccharide biosynthesis protein	1	1	1	1	1	1	1	1	1	1
SP_0350	cpsE; capsular polysaccharide biosynthesis protein	1	1	1	1	1	1	1	1	1	1
SP_0117	pspA; pneumococcal surface protein A	1	1	1	1	1	1	1	1	1	1
SP_0377	cbpC; choline-binding protein C	1	1	1	1	1	1	1	1	1	1
SP_0378	cbpJ; choline-binding protein J	1	1	1	1	1	1	1	1	1	1
SP_0390	cbpG; choline-binding protein G	1	1	1	1	1	1	1	1	1	1
SP_0391	cbpF; choline-binding protein F	1	1	1	1	1	1	1	1	1	1
SP_0533	blpK; bacteriocin associated protein	1	1	1	1	1	1	1	1	1	1
SP_0545	blpY; Immunity protein	1	1	1	1	1	1	1	1	1	1
SP_0641	prtA; protective antigen A	1	1	1	1	1	1	1	1	1	1
SP_1638	PsaR; transcriptional regulator	1	1	1	1	1	1	1	1	1	1
SP_0966	pavA; adherence and virulence protein A	1	1	1	1	1	1	1	1	1	1
SP_1923	pln; pneumolysin	1	1	1	1	1	1	1	1	1	1
SP_1937	lytA; autolysin	1	1	1	1	1	1	1	1	1	1
SP_2190	chpA; choline-binding protein A	1	1	1	1	1	1	1	1	1	1
SP_2201	chpD; choline-binding protein D	1	1	1	1	1	1	1	1	1	1
SP_0457	bacA; bacitracin resistance protein	1	1	1	1	1	1	1	1	1	1
SP_0615	fibA; beta-lactam resistance factor	1	1	1	1	1	1	1	1	1	1
SP_1673	penA; penicillin-binding protein 2B	1	1	1	1	1	1	1	1	1	1
SP_0369	penicillin-binding protein 1A	1	1	1	1	1	1	1	1	1	1
SP_2099	penicillin-binding protein 1B	1	1	1	1	1	1	1	1	1	1
SP_2010	penicillin-binding protein 2A	1	1	1	1	1	1	1	1	1	1
SP_0336	penicillin-binding protein 2X	1	1	1	1	1	1	1	1	1	1
SP_0616	beta-lactam resistance factor	1	1	1	1	1	1	1	1	1	1
SP_0798	ciaR; DNA-binding response regulator	1	1	1	1	1	1	1	1	1	1
SP_0799	ciaH; sensor histidine kinase	1	1	1	1	1	1	1	1	1	1
SP_0972	pmrA; multi-drug resistance efflux pump	1	1	1	1	1	1	1	1	1	1
SP_0461	transcriptional regulator	0	0	1	1	1	1	0	0	0	0

All *S. pneumoniae* loci number (sp_#) are according to the annotation of reference strain TIGR4. 1 indicates presence and 0 indicates absence of the gene from the pneumococcal isolates

**Table 4:** The total number of non-synonymous SNPs for each pneumococcal isolate

Isolate	No. of SNPs	SNPs in protein coding
SPS1	3600	3180
SPS2	2847	2522
SPS3	6297	5611
SPS4	6339	5668
SPS5	6359	5675
SPS6	6411	5731
SPS7	6704	5998
SPS8	6440	5761
SPS9	6452	5776
SPS10	6548	5875

**Table 5:** Non-synonymous SNPs among *S. pneumoniae* isolates found in genes associated with virulence, antibiotic resistance, and other regulatory functions

Locus name^a^	Gene name and/or description	No. of SNPs in Pneumococcal isolates									
		SPS01	SPS02	SPS03	SPS04	SPS05	SPS06	SPS07	SPS08	SPS09	SPS10
SP_0041	blpU; bacteriocin	1	1	1	1	1	1	1	1	–	2
SP_0346	cpsA; capsular polysaccharide biosynthesis protein	7	5	4	4	4	4	8	8	8	8
SP_0347	cpsB; capsular polysaccharide biosynthesis protein	1	2	–	–	–	–	1	1	1	5
SP_0348	cpsC; capsular polysaccharide biosynthesis protein	1	3	3	3	3	3	2	2	2	6
SP_0349	cpsD; capsular polysaccharide biosynthesis protein	7	–	11	11	12	12	5	5	9	–
SP_0350	cpsE; capsular polysaccharide biosynthesis protein	–	–	–	–	–	–	–	–	–	–
SP_0351	cpsF; capsular polysaccharide biosynthesis protein	–	–	–	–	–	–	–	–	–	–
SP_0352	cpsG; capsular polysaccharide biosynthesis protein	–	–	–	–	–	–	–	–	–	–
SP_0353	cpsH; capsular polysaccharide biosynthesis protein	–	–	–	–	–	–	–	–	–	–
SP_1837	capsular polysaccharide biosynthesis protein	4	3	–	–	–	–	3	3	4	4
SP_0117	pspA; pneumococcal surface protein A	2	–	4	2	3	4	5	5	4	3
SP_0377	cbpC; choline-binding protein C	6	3	5	5	5	5	1	1	–	1
SP_0930	cbpE; choline-binding protein E	7	8	12	11	13	12	3	3	11	5
SP_0378	cbpJ; choline-binding protein J	1	3	2	2	2	2	1	1	–	1
SP_0390	cbpG; choline-binding protein G	3	6	4	4	2	4	4	4	3	1
SP_0391	cbpF; choline-binding protein F	5	6	–	–	–	–	–	–	–	–
SP_0069	cbpI; choline-binding protein I	–	–	–	–	–	–	–	–	–	–
SP_0533	blpK; bacteriocin associated protein	–	–	–	–	–	–	–	–	–	–
SP_0545	blpY; Immunity protein	2	3	3	3	2	2	2	2	–	1
SP_0641	prtA; protective antigen A	16	14	13	12	12	13	14	14	9	13
SP_0730	spxB; pyruvate oxidase	1	1	–	–	–	–	–	–	1	–
SP_1002	lmb; adhesion lipoprotein	–	–	–	–	–	–	–	–	–	3
SP_1003	Conserved hypothetical protein	7	6	5	3	4	5	8	8	4	4
SP_1207	xseA; exodeoxyribonuclease VII, large subunit	–	–	–	–	–	–	–	–	–	–
SP_1923	pln; pneumolysin	1	1	–	–	–	–	2	2	2	3
SP_1937	lytA; autolysin	1	4	1	1	1	1	1	1	1	–
SP_2190	chpA; choline-binding protein A	6	11	9	10	7	8	2	2	4	9
SP_2201	chpD; choline-binding protein D	3	4	4	4	4	4	6	6	3	8
SP_2239	htrA; serine protease	–	1	–	–	–	–	1	1	2	2
SP_2240	spoJ; homologous to sporulation protein	1	–	2	2	2	2	1	1	1	1
SP_0457	bacA; bacitracin resistance protein	–	–	–	–	–	–	–	–	–	–
SP_0615	fibA; beta-lactam resistance factor	–	1	1	2	2	1	2	1	1	4
SP_1673	penA; penicillin-binding protein 2B	1	6	3	1	5	5	2	2	3	2
SP_0369	penicillin-binding protein 1A	3	2	1	2	1	1	2	2	–	3
SP_2099	penicillin-binding protein 1B	3	2	4	4	4	4	3	3	4	4
SP_2010	penicillin-binding protein 2A	4	5	5	5	5	5	1	1	7	5
SP_0336	penicillin-binding protein 2X	1	1	3	2	1	2	2	2	4	3
SP_0616	beta-lactam resistance factor	2	1	1	2	2	1	4	4	3	2
SP_1075	CpoA; glycosyl transferase	1	1	1	1	1	1	–	–	1	1
SP_0798	ciaR; DNA-binding response regulator	–	1	–	1	–	–	–	–	–	–
SP_0799	ciaH; sensor histidine kinase	–	–	–	–	–	–	1	1	–	–
SP_2084	pstS; phosphate ABC transporter, phosphate-bind	–	–	–	–	–	–	–	–	–	1
SP_2085	pstC; phosphate ABC transporter, permease protein	–	2	3	3	3	3	2	2	1	1
SP_2086	pstA; phosphate ABC transporter, permease protein	–	–	–	–	–	–	–	–	–	–
SP_2087	pstB; phosphate ABC transporter, ATP-binding protein	3	3	1	1	1	1	–	–	1	–
SP_1890	amiC; oligopeptide ABC transporter permease	2	3	3	3	3	3	4	4	4	2
SP_0010	beta-lactamase class C	4	–	1	1	1	1	–	–	1	1
SP_0314	hyaluronate lyase	14	14	15	15	15	15	16	16	10	14
SP_0071	metalloprotease ZmpC	–	–	–	–	–	–	–	–	–	–
SP_1650	manganese ABC transporter substrate-binding lipoprotein	1	1	1	1	1	1	1	1	1	1
SP_1687	neuraminidase B	7	7	8	4	4	8	5	5	5	4
SP_0357	UDP-N-acetyl glucosamine 2-epimerase CpsI	–	–	–	–	–	–	–	–	–	–
SP_0358	capsular polysaccharide biosynthesis protein CpsJ	–	–	–	–	–	–	–	–	–	–
SP_0359	capsular polysaccharide biosynthesis protein CpsK	–	–	–	–	–	–	–	–	–	–
SP_0360	UDP-N-acetyl glucosamine 2-epimerase CpsL	–	–	–	–	–	–	–	–	–	–
SP_0463	cell wall surface anchor protein	–	–	1	1	1	1	–	–	–	–
SP_0461	transcriptional regulator	–	–	–	–	1	–	–	–	–	–
SP_0468	sortase	–	–	–	–	–	–	–	–	–	–
SP_0965	endo-beta-N-acetylglucosaminidase LytB	2	2	2	2	2	2	1	1	1	2
SP_1783	DNA mismatch repair protein MutT	–	–	1	1	1	1	–	–	–	1
SP_0173	DNA mismatch repair protein HexB	–	–	–	–	–	–	–	–	–	–
SP_2218	MreC; rod shape-determining protein	–	–	–	–	–	–	–	–	–	–
SP_rrnaA23S	23S ribosomal RNA; K01980 23S ribosomal RNA	–	–	–	–	–	–	–	–	–	–
SP_rrnaB23S	23S ribosomal RNA; K01980 23S ribosomal RNA	–	–	–	–	–	–	–	–	–	–
SP_rrnaC23S	23S ribosomal RNA; K01980 23S ribosomal RNA	–	–	–	–	–	–	–	–	–	–
SP_rrnaD23S	23S ribosomal RNA; K01980 23S ribosomal RNA	–	–	–	–	–	–	–	–	–	–

^a^All *S. pneumoniae* loci number (sp_#) are according to the annotation of reference strain TIGR4

**Table 6:** Unique non-synonymous single nucleotide polymorphisms (SNPs) associated with penicillin binding proteins (PBPs) and other virulent genes found in all ten pneumococcal isolates isolates

Locus Name	Putative Identification	Reference Position	TIGR4	SNP	Pneumococcal isolate	Amino Acid Change
SP_0346	cpsA; capsular polysaccharide biosynthesis protein	320,234	C	T	SPS7, SPS8, SPS10	A53V
		320,204	C	T	SPS7, SPS8	A43V
		320,872	C	T	SPS10	P266S
		321,451	G	A	SPS10	V459 M
		320,657	C	T	SPS9, SPS10	S194 L
		321,460	A	G	SPS2	I462V
		321,485	T	C	SPS7, SPS8	V470A
		320,710	A	G	SPS1	T212A
		322,321	A	G	SPS10	K20E
		322,360	G	A	SPS2	G33S
		323,191	A	C	SPS1, SPS7, SPS8	N76H
SP_1837	capsular polysaccharide biosynthesis protein	1,746,914	T	C	SPS1, SPS9, SPS10	K212R
SP_0117	pspA; pneumococcal surface protein A	118,490	C	T	SPS9	T23 M
		120,431	C	A	SPS7, SPS8	A670D
		118,496	A	C	SPS7, SPS8, SPS10	Q25P
SP_0799	ciaH; sensor histidine kinase ClaH	753,163	C	G	SPS7, SPS8	H180D
SP_1923	pln; pneumolysin	1,832,851	G	A	SPS9	T154 M
SP_0369	penicillin-binding protein 1A	347,479	C	T	SPS2	E512K
		348,706	T	A	SPS2	T103S
SP_2099	penicillin-binding protein 1B	2,006,807	A	G	SPS10	V787A
SP_2010	penicillin-binding protein 2A	1,917,863	T	C	SPS9, SPS10	E17G
		1,916,459	T	G	SPS9	A485E
		1,916,166	C	T	SPS9	A583T
SP_1673	penA; penicillin-binding protein 2B	1,573,212	C	A	SPS9	L609F
		1,574,933	C	T	SPS3	V36I
		1,573,493	C	A	SPS2	A516S
		1,574,288	C	A	SPS2, SPS3	A251S
		1,574,461	G	A	SPS2	A193V
SP_0336	penicillin-binding protein 2X	308,341	G	A	SPS9	D488N
		307,393	G	A	SPS2, SPS3	A172T
		309,113	C	A	SPS3	T745 K

**Fig. 3:**
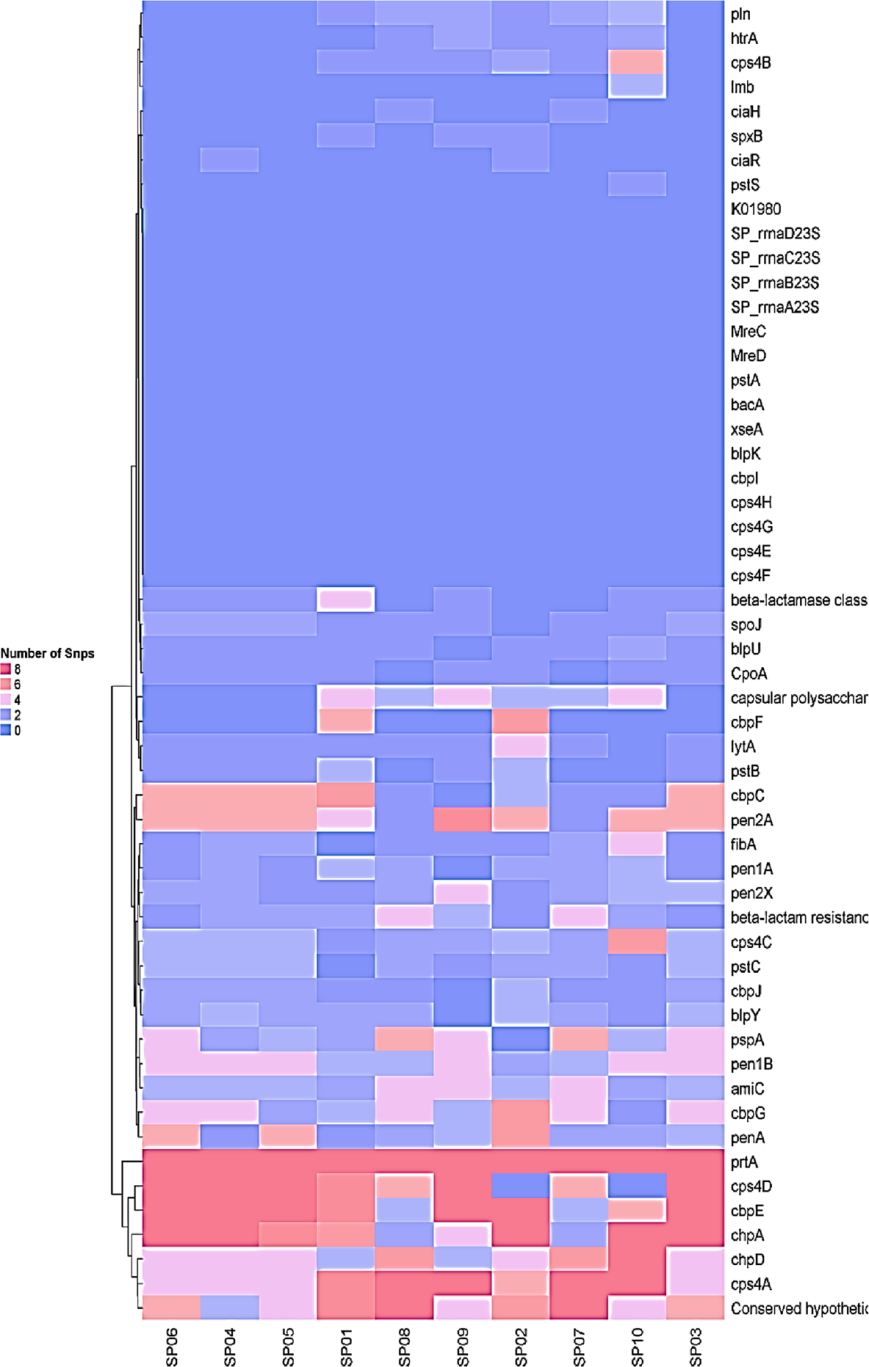
Heatmap represents the number of Non-synonymous SNPs from *S. pneumoniae* isolates from antibiotic-resistance genes

**Fig. 4:**
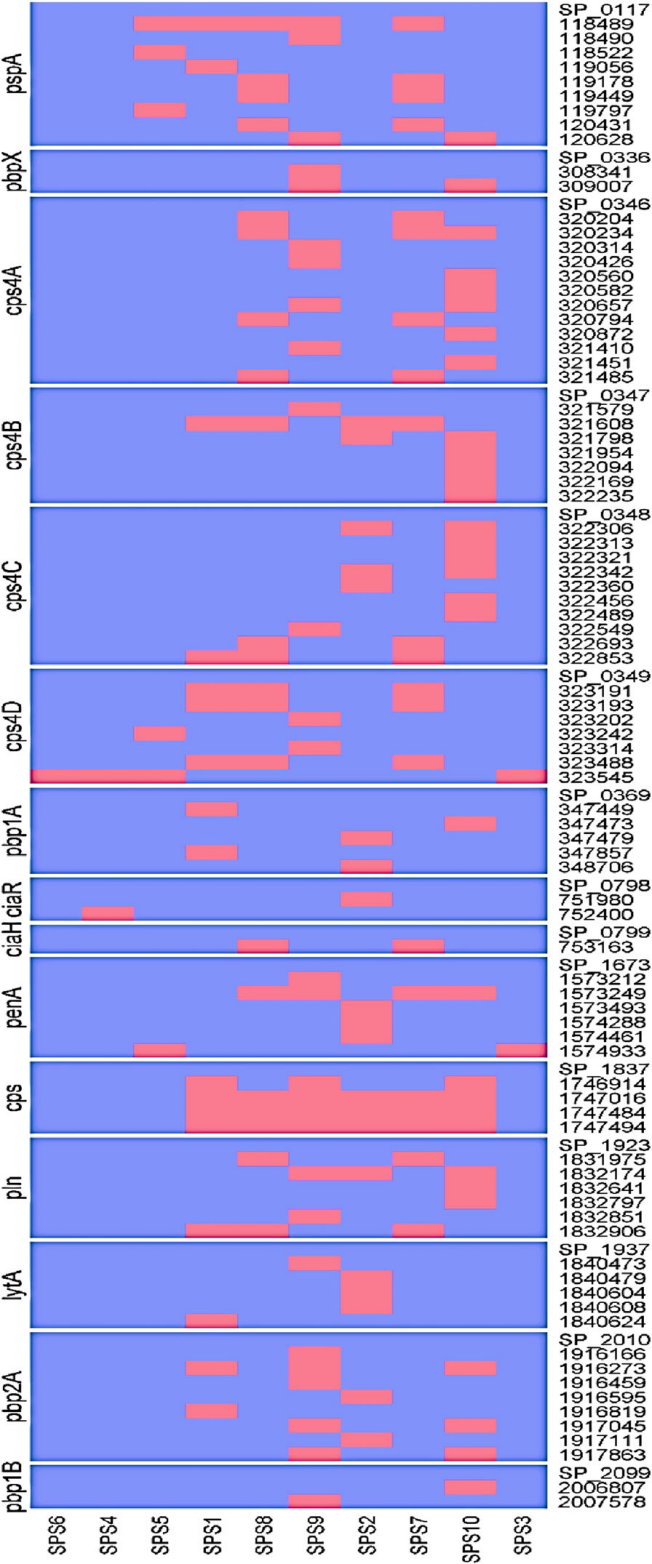
Heatmap represents the presence and absence of SNPs (numbers on the right side) in some of virulent genes in all ten isolates. Annotation on the right side of the heat map is the gene names. (Blue color represents absence and pink color represents presence)

The numbers of non-synonymous SNPs corresponding to the selected genes essential for bacterial survival and virulence based on previous literatures [37–39] in each isolate are presented in Table 5. Figure 3 represents the number of non-synonymous SNPs that are in genes associated with virulence and antibiotics resistance. Resistant isolates to all four antibiotics have higher numbers of SNPs associated with virulent genes than susceptible isolates (SPS4 and SPS6), suggesting that the presence of certain SNPs could be more related to drug-resistance (Fig. 4). Genes encoding capsular polysaccharide (CPS) biosynthesis proteins Cps4E, Cps4F, Cps4G, and Cps4H did not possess any mutations amongst all the pneumococcal isolates (Table 5).

### Phylogenetic analysis of *S. pneumoniae* isolates

**Fig. 5:**
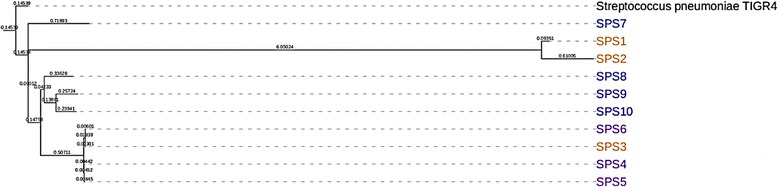
Parsimony tree with respect to reference and ten isolates was generated from kSNP3 pipeline. Parsimony tree is consensus tree based all of the SNPs identified between the reference and isolates. Branch lengths are expressed in terms of changes per number of SNP

A parsimony tree with respect to reference and 10 isolates was generated from the kSNP3 pipeline, parsimony tree is consensus tree based on all of the SNPs identified between the reference genome TIGR4 and the 10 isolates. Branch lengths are expressed in terms of changes per number of SNPS. Our result showed that isolates SPS3, SPS4, SPS5, and SPS6 were closest to each other, while isolates SPS8, SPS9, and SPS10 were closely related to each other. All these eight isolates formed one clade. On the other hand isolates SPS1, SPS2, and SPS7 were closely related to each other (Fig. 5).

## Discussion

WGS has become an essential tool to elucidate the mechanisms used by bacteria to resist various antibiotics. In the present study, we have investigated the genomic variations and mutations among genes associated with virulence and antibiotic resistance in 10 clinical isolates of *S. pneumoniae* selected based on their susceptibility profiles against four antibiotics. Using WGS technique, the full genomic sequences of the 10 isolates were compared to that of the *S. pneumoniae* reference genome TIGR4.

Whole genome sequencing of the 10 isolates has revealed a high degree of sequence conservation between the pneumococcal isolates regardless of their susceptibility to antibiotics. This high sequence similarity of the isolates could possibly be explained by the low number of isolates and also by the fact that all the isolates had been collected from the same hospital. Nevertheless, these results are in agreement with previous studies showing that closely related isolates may possess different levels of resistance to antibiotics [40]. The genes known to be involved in antibiotic resistance are well conserved among all the 10 isolates; however, we were able to identify many mutations that differentiate resistant form susceptible isolates.

Our results showed that the majority of the SNPs occur in the resistant strains rather than the susceptible strains (Fig. 3). For instance, penicillin-resistant isolates showed a greater number of SNPs (3301) compared to susceptible isolates (281 SNPs). Figure 3 reveals that the highest numbers of SNPs among all the isolates are present in prtA, CpsD, CbpE, CbpA, CbpD, and CpsA. These genes could play a role in pneumococcal resistance to antibiotics. We were able to identify 90 non-synonymous SNPs associated with the essential genes of the resistant isolates, and some of them have reappeared in more than one resistant isolate, while none of them have occurred in susceptible strains (Additional file 3). Figure 4 shows that resistant isolates possess a higher number of SNPs associated with virulent genes than the susceptible isolates (SPS4 and SPS6). These results suggest that the presence of particular SNPs could play a role in conferring resistance to antibiotics. Out of these 90 SNPs, 31 were unique and found in penicillin binding proteins (PBP1A, PBP1B, PBP2A, PBP2B, and PBP2X), virulent genes (pneumolysin and PspA), sensor histidine kinase (ciaH), and CpsA; capsular polysaccharide biosynthesis protein (Table 6). However, the role of these SNPs in antibiotic resistance need to be investigated as some of them especially those related to penicillin binding proteins were also present in isolates SPS8 and SPS9 that are susceptible to penicillin.

In our study, we observed that genes encoding capsular polysaccharide biosynthesis proteins CpsE, CpsF, CpsG, and CpsH did not contribute to antibiotic resistance in all the resistant-types (Table 5). On the other hand, our results revealed that several novel mutations are present within capsular biosynthesis genes CpsA, CpsB, CpsC, and CpsD associated with resistant isolates (Table 3). The synthesis of capsular polysaccharides is regulated by a set of genes located at the same locus (*cps*) between *dexB* and *aliA*. Except for serotypes 3 and 37, the first four genes of *cps* locus (*CpsA-D*) are common in all pneumococcal serotypes. These four genes encode proteins that affect the level of CPS expression [41]. Although CpsA has no impact on the transcription of CPS in *S. pneumoniae*, a mutant of pneumococcus lacking CpsA has been shown to produce a reduced level of CPS [42]. CpsB is a manganese-dependent phosphotyrosine-protein phosphatase; it has been shown that CpsB is necessary for the dephosphorylation of CpsD. Mutants with CpsB deletions tend to have an increased level of phosphorylated CpsD, which leads to a significant decrease in production of CPS [43]. CpsC is a membrane protein required for CpsD tyrosine autophosphorylation. A novel role for CpsC in the attachment of CPS to the pneumococcal cell wall has been identified recently [44]. CpsD is an auto-phosphorylating tyrosine kinase. Mutations in CpsD affecting the ATP-binding domain eliminate CPS production in *S*. *pneumococcus*. Therefore, the capsular genes CpsB, CPsC and CpsD work together to regulate CPS biosynthesis [43, 44].

*S. pneumoniae* resists penicillin and other β-lactams by altering PBPs, the main enzymes involved in the final stage of cell wall synthesis. Six PBPs have been identified in pneumococcus PBP1a, 1b, 2×, 2a, 2b, and 3 [15]*.* Mutations in three of the PBPs (PBP2b, PBP2x, and PBP1a) have the most significant effect on β-lactams resistance. Several groups have reported mutations in genes encoding PBPs [45–47]. The MIC levels for isolates SPS1 and SPS2 were 2 μg/ml for penicillin and 1 μg/ml for cefotaxime. On the other hand, SPS7 and SPS10 showed the same level of resistance toward penicillin but an increased resistance for cefotaxime (2 μg/ml). Through our analysis, we were able to identify a non-synonymous SNP (G597E) in both SPS7 and SPS10 associated with penicillin binding protein PBP2B. The same SNP was found in isolates SPS8 and SPS9, and both of these two isolates showed high MICs toward cefotaxime (> 8 μg/ml and 8 μg/ml, respectively) (Additional file 3). However, our results showed that all the pneumococcal isolates regardless of their sensitivity to penicillin possess same mutations in the genes encoding penicillin-binding proteins, confirming previous reports that showed that these proteins are not the only determinants of penicillin resistance [48]. Furthermore, we were able to identify a unique mutation (H180D) in the sensor histidine kinase gene (ciaH) in resistant isolates only (Additional file 3). Mutations in ciaH increase resistance to β-lactams, as this gene is involved in the biosynthesis of cell wall components [49].

The phylogenetic relationships among different clinical isolates of *S. pneumonaie* were examined using the parsimony tree based on SNPs from whole genome sequencing. From the results, we observed that isolates SPS1 and SPS2 were clustered in one clade; isolates SPS8, SPS9, and SPS10 grouped in one clade; and isolates SPS4, SPS5 and SPS6 clustered in different clade (Fig. 5). These results are consistent with the MIC profile of the 10 isolates (Table 2). The observations that pneumococcal isolates with similar MIC profile were grouped together in a phylogenetic tree suggest that they possess common mutations and were probably originated from the common clone. It is possible that these strains could have evolved and acquired mutations in a similar manner due to selection pressures. Surprisingly, our results revealed that resistant isolate SPS3 was closely related to the susceptible isolates SPS4, SPS5, and SPS6. Among all the resistant isolates, SPS10 showed the highest number of SNPs (34 SNPs) (Table 3). On the other hand, SPS3 was the least resistant isolate having non-synonymous SNPs compared to other resistant isolates. Moreover, three non-synonymous SNPs (I178T, V22A, and T19A) were common among all the resistant isolates except isolate SPS3 (Additional file 3). This finding suggests that SPS3 could resist antibiotics using a unique mechanism as compared to other resistant isolates. The high phylogenetic relatedness among the clinical pneumococcal isolates with similar MIC profile is related to the specific SNPs in the mutated genes. The presence of identical uncommon mutations, as well as certain genes in the grouped isolates in the phylogenetic tree, is indicative of a single cluster of strains circulating in the population. For instance, the mutations S29A in cpsB, H197L in cpsC, M79I in cpsD, and Q136K in Ply were all found in isolates SPS1, SPS7, and SPS8 (Table 4). All three isolates are closely related to each other in the phylogenetic tree (Fig. 5). Similarly, certain genes such as SP_0461, SP_0463, SP_0357, and SP_1765 were only found in pneumococcal isolates SPS3, SPS4, SPS5, and SPS6 (Additional file 1).

## Conclusion

In conclusion, this study compared the genomic sequences of 10 pneumococcal isolates with different susceptibility to multiple antibiotics. The high degree of sequence conservation and the presence of the same SNPs especially those related to genes involved in β-lactam resistance in both sensitive and resistant isolates, makes it a difficult task to identify distinct mechanisms of resistance that differentiate strains with different drug-sensitivities, and that antibiotic resistance cannot be only linked to the presence of certain genes. These results are in agreement with previous assumptions that bacterial virulence is the result of a gathering of pathogenicity-related genes that interact in various combinations [50] and that multi-drug resistance could be a result of combinations of mutations that lead to overexpression of several multi-drug efflux pumps; outer membrane porins, β-lactam acylases and enzymes and structural components involved in peptidoglycan stability (targets of β-lactams); gyrase mutations; and aminoglycoside phosphotransferases and –acetylases [40]. We were able to identify unique SNPs associated with virulent genes that could have a possible role in resistance to various antibiotics. To confirm these results future studies on virulence gene knockouts are needed to link the role of antibiotic resistance with these genes. This study was also limited by the relatively small number of isolates included in the analysis. Moreover, all resistant genes have yet to be subjected to individual mutational analysis. This can be achieved by introducing the SNPs on the resistant genes by site-directed mutagenesis and further expression analysis. The development of bacterial resistance towards antibiotics is a complex mechanism and multiple genetic alterations such as addition/deletion of specific genes, mutations, or a combination of both could be involved in the process. Whole genome sequencing can be utilised in conjunction with current epidemiological studies, diagnostic assays, and antimicrobial susceptibility tests to understand the genetic variation and pathogen biology of “high-risk” bacteria. It is also important to note that t.

## Additional files

Additional file 1: Assembly and gene content of all the ten pneumococcal clinical isolates. (XLSX 288 kb)

Additional file 2: Full list of non-synonymous single nucleotide polymorphisms (SNPs) in all ten pneumococcal clinical isolates. (XLSX 8827 kb)

Additional file 3: Conserved non-synonymous single nucleotide polymorphisms (SNPs) associated with penicillin binding proteins (PBPs) and other virulent genes found in resistant isolates. (DOCX 29 kb)
