# Supplementary material for: Comparative genomic analysis of ten clinical Streptococcus pneumoniae collected from a Malaysian hospital reveal 31 new unique drug-resistant SNPs using whole genome sequencing
Source: J Biomed Sci. 2018 Feb 15;25:15. doi: 10.1186/s12929-018-0414-8 (PMC5815235; doi:10.1186/s12929-018-0414-8)
Supplement: Supplementary file 3 — Conserved non-synonymous single nucleotide polymorphisms (SNPs) associated with penicillin binding proteins (PBPs) and other virulent genes found in resistant isolates. (DOCX 29 kb) [file 12929_2018_414_MOESM3_ESM.docx]

**Additional file 3.** Conserved non-synonymous single nucleotide polymorphisms (SNPs) associated with penicillin binding proteins (PBPs) and other virulent genes found in resistant isolates.

| **Locus Name** | **Putative Identification** | **Reference**  **Position** | **TIGR4** | **SNP** | **Pneumococcal isolate** | **Amino Acid Change** |
| --- | --- | --- | --- | --- | --- | --- |
| SP_0346 | cpsA; capsular polysaccharide biosynthesis protein | 320234 | C | T | SPS7, SPS8, SPS10 | A53V |
|  |  | 320204 | C | T | SPS7, SPS8 | A43V |
|  |  | 320872 | C | T | SPS10 | P266S |
|  |  | 321451 | G | A | SPS10 | V459M |
|  |  | 320582 | C | T | SPS10 | A169V |
|  |  | 320657 | C | T | SPS9, SPS10 | S194L |
|  |  | 320560 | A | G | SPS10 | N162D |
|  |  | 321410 | T | C | SPS9 | M445T |
|  |  | 320314 | G | C | SPS1, SPS9 | V80L |
|  |  | 321460 | A | G | SPS2 | I462V |
|  |  | 321485 | T | C | SPS7, SPS8 | V470A |
|  |  | 320101 | C | A | SPS2 | R9S |
|  |  | 320710 | A | G | SPS1 | T212A |
| SP_0347 | cpsB; capsular polysaccharide biosynthesis protein Cps4B | 321798 | A | G | SPS2, SPS10 | E92G |
|  |  | 321954 | A | G | SPS10 | E144G |
|  |  | 322094 | G | A | SPS10 | D191N |
|  |  | 322169 | C | T | SPS10 | L216F |
|  |  | 322235 | G | A | SPS10 | V238I |
|  |  | 321579 | G | T | SPS9 | R19I |
|  |  | 321608 | T | G | SPS1, SPS2, SPS7, SPS8 | S29A |
| SP_0348 | cpsC; capsular polysaccharide biosynthesis protein | 322306 | G | T | SPS2, SPS10 | V15F |
|  |  | 322313 | G | C | SPS10 | S17T |
|  |  | 322321 | A | G | SPS10 | K20E |
|  |  | 322342 | A | T | SPS2, SPS10 | I27L |
|  |  | 322456 | C | T | SPS10 | P65S |
|  |  | 322489 | A | T | SPS10 | T76S |
|  |  | 322853 | A | T | SPS1, SPS7, SPS8 | H197L |
|  |  | 322360 | G | A | SPS2 | G33S |
|  |  | 322693 | G | A | SPS7, SPS8 | E144K |
|  |  | 322549 | G | A | SPS9 | V96I |
| SP_0349 | cpsD; capsular polysaccharide biosynthesis protein | 323314 | G | A | SPS9 | V117I |
|  |  | 323202 | G | A | SPS9 | M79I |
|  |  | 323488 | G | A | SPS1, SPS7, SPS8 | V175I |
|  |  | 323191 | A | C | SPS1, SPS7, SPS8 | N76H |
|  |  | 323193 | T | A | SPS1, SPS7, SPS8 | N76K |
|  |  | 323416 | A | G | SPS1, SPS7, SPS8, SPS9 | I151V |
| SP_1837 | capsular polysaccharide biosynthesis protein | 1746914 | T | C | SPS1, SPS9, SPS10 | K212R |
|  |  | 1747016 | A | G | SPS1, SPS2, SPS7, SPS8, SPS9, SPS10 | I178T |
|  |  | 1747484 | A | G | SPS1, SPS2, SPS7, SPS8, SPS9, SPS10 | V22A |
|  |  | 1747494 | T | C | SPS1, SPS2, SPS7, SPS8, SPS9, SPS10 | T19A |
| SP_0117 | pspA; pneumococcal surface protein A | 118489 | A | G | SPS1, SPS7, SPS8, SPS9 | T23A |
|  |  | 118490 | C | T | SPS9 | T23M |
|  |  | 120628 | A | G | SPS9, SPS10 | K736E |
|  |  | 120431 | C | A | SPS7, SPS8 | A670D |
|  |  | 119178 | A | C | SPS7, SPS8 | K252N |
|  |  | 119449 | A | C | SPS7, SPS8 | K343Q |
|  |  | 119056 | T | G | SPS1 | Y212D |
|  |  | 118496 | A | C | SPS7, SPS8, SPS10 | Q25P |
| SP_0799 | ciaH; sensor histidine kinase ClaH | 753163 | C | G | SPS7, SPS8 | H180D |
| SP_1923 | pln; pneumolysin | 1832851 | G | A | SPS9 | T154M |
|  |  | 1832174 | T | C | SPS2, SPS9, SPS10 | N380D |
|  |  | 1832641 | T | C | SPS10 | K224R |
|  |  | 1832797 | G | A | SPS10 | T172I |
|  |  | 1831975 | G | A | SPS7, SPS8 | P446L |
|  |  | 1832906 | G | T | SPS1, SPS7, SPS8 | Q136K |
| SP_1937 | lytA; autolysin | 1840479 | T | G | SPS2 | L295I |
|  |  | 1840604 | A | T | SPS2 | E253V |
|  |  | 1840608 | C | T | SPS2 | N252D |
|  |  | 1840473 | A | G | SPS9 | P297S |
|  |  | 1840624 | A | C | SPS1 | D246E |
| SP_0369 | penicillin-binding protein 1A | 347449 | C | T | SPS1 | A522T |
|  |  | 347857 | C | T | SPS1 | V386I |
|  |  | 347479 | C | T | SPS2 | E512K |
|  |  | 348706 | T | A | SPS2 | T103S |
|  |  | 347473 | C | G | SPS10 | E514Q |
| SP_2099 | penicillin-binding protein 1B | 2006807 | A | G | SPS10 | V787A |
|  |  | 2007578 | T | G | SPS9 | E530A |
| SP_2010 | penicillin-binding protein 2A | 1917863 | T | C | SPS9, SPS10 | E17G |
|  |  | 1917045 | T | C | SPS9, SPS10 | T290A |
|  |  | 1916273 | C | T | SPS1, SPS9, SPS10 | S547N |
|  |  | 1916459 | T | G | SPS9 | A485E |
|  |  | 1916166 | C | T | SPS9 | A583T |
|  |  | 1917111 | G | T | SPS2 | Q268K |
|  |  | 1916595 | C | T | SPS2 | D440N |
|  |  | 1916819 | A | G | SPS1 | F365S |
| SP_1673 | penicillin-binding protein 2B | 1573249 | C | T | SPS7, SPS8, SPS9, SPS10 | G597E |
|  |  | 1573212 | C | A | SPS9 | L609F |
|  |  | 1574933 | C | T | SPS3 | V36I |
|  |  | 1573493 | C | A | SPS2 | A516S |
|  |  | 1574288 | C | A | SPS2, SPS3 | A251S |
|  |  | 1574461 | G | A | SPS2 | A193V |
| SP_0336 | penicillin-binding protein 2X | 309007 | C | T | SPS9, SPS10 | L710F |
|  |  | 308341 | G | A | SPS9 | D488N |
|  |  | 307393 | G | A | SPS2, SPS3 | A172T |
|  |  | 309113 | C | A | SPS3 | T745K |
| SP_0798 | ciaR; DNA-binding response regulator | 751980 | G | A | SPS2 | V7I |
| SP_0377 | cbpC; choline-binding protein C | 356412 | G | A | SPS1 | G156S |
|  |  | 355972 | A | C | SPS2 | Q9P |
|  |  | 355974 | G | A | SPS2 | V10I |
|  |  | 356806 | C | T | SPS2 | S287L |
|  |  | 356182 | C | A | SPS7, SPS8 | P79H |
|  |  | 356044 | A | G | SPS10 | R33Q |
